# Supplementary material for: A Humanized Yeast Phenomic Model of Deoxycytidine Kinase to Predict Genetic Buffering of Nucleoside Analog Cytotoxicity
Source: Genes (Basel). 2019 Sep 30;10(10):770. doi: 10.3390/genes10100770 (PMC6826991; doi:10.3390/genes10100770)
Supplement: Supplementary file 1 [file genes-10-00770-s001.zip › Additional_Files/Additional_File7_GOTermHeatmaps.zip!/Additional_File7_GOTermHeatmaps/ribosomal_large_subunit_biogenesis.pdf]

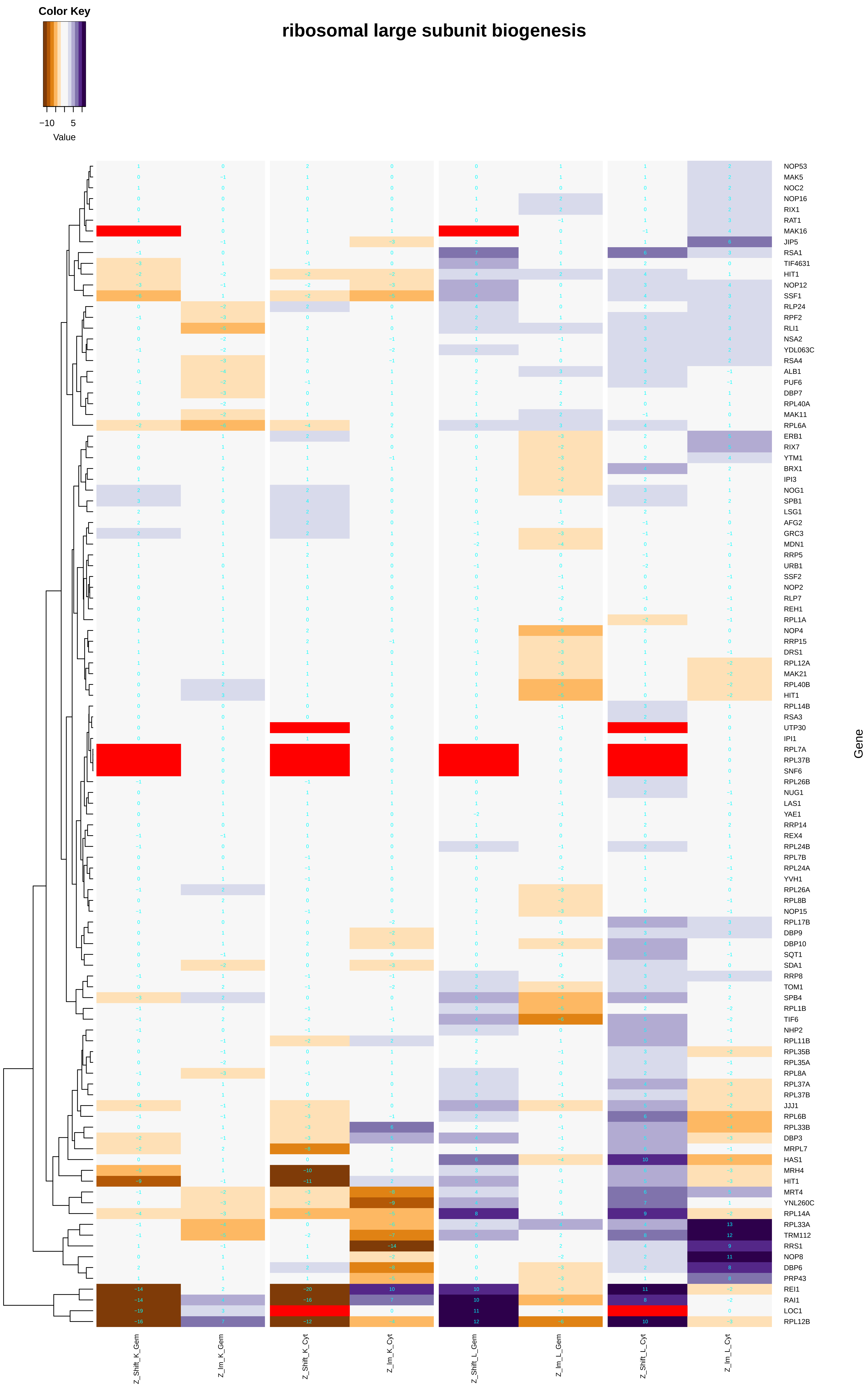
ribosomal large subunit biogenesis
Color Key
−10
5
Value
NOP53
MAK5
NOC2
NOP16
RIX1
RAT1
MAK16
JIP5
RSA1
TIF4631
HIT1
NOP12
SSF1
RLP24
RPF2
RLI1
NSA2
YDL063C
RSA4
ALB1
PUF6
DBP7
RPL40A
MAK11
RPL6A
ERB1
RIX7
YTM1
BRX1
IPI3
NOG1
SPB1
LSG1
AFG2
GRC3
MDN1
RRP5
URB1
SSF2
NOP2
RLP7
REH1
RPL1A
NOP4
RRP15
DRS1
RPL12A
MAK21
RPL40B
HIT1
RPL14B
RSA3
UTP30
IPI1
RPL7A
RPL37B
SNF6
RPL26B
NUG1
LAS1
YAE1
RRP14
REX4
RPL24B
RPL7B
RPL24A
YVH1
RPL26A
RPL8B
NOP15
RPL17B
DBP9
DBP10
SQT1
SDA1
RRP8
TOM1
SPB4
RPL1B
TIF6
NHP2
RPL11B
RPL35B
RPL35A
RPL8A
RPL37A
RPL37B
JJJ1
RPL6B
RPL33B
DBP3
MRPL7
HAS1
MRH4
HIT1
MRT4
YNL260C
RPL14A
RPL33A
TRM112
RRS1
NOP8
DBP6
PRP43
REI1
RAI1
LOC1
RPL12B
Gene
Z_Shift_K_Gem
Z_Im_K_Gem
Z_Shift_K_Cyt
Z_Im_K_Cyt
Z_Shift_L_Gem
Z_Im_L_Gem
Z_Shift_L_Cyt
Z_Im_L_Cyt
